# Case-control analysis of hip fractures with concurrent benzodiazepine and opioid use and surgery class at a single teaching institution

**DOI:** 10.1097/MD.0000000000039743

**Published:** 2024-09-13

**Authors:** Andrew Z. Coskey, Ernst J. Nicanord, Margaret A. Goodwin, Haris Vakil, Daniel C. Jupiter, John C. Hagedorn II, Namita Bhardwaj

**Affiliations:** a Department of Orthopaedic Surgery and Rehabilitation, The University of Texas Medical Branch, Galveston, TX; b Department of Family Medicine, The University of Texas Medical Branch, Galveston, TX; c Department of Preventive Medicine and Population Health, The University of Texas Medical Branch, Galveston, TX.

**Keywords:** alcohol, benzodiazepines, femoral neck fractures, fracture displacement, hip fractures, opioids

## Abstract

In this study, we analyze the relationship between polypharmacy and surgical treatment in a population at a single teaching institution. The design of the study is a case-control analysis of hip fractures. The setting is at a single teaching institution located in Galveston, Texas, USA. Over a 5-year period, we conducted a retrospective review of patients within our medical record who underwent surgery for a hip fracture, identified by current procedural terminology codes 27235 and 27236. Our primary variable was a prescription of opioids, benzodiazepines, or both 30 days preoperatively and surgery performed. The main outcome measures were prescription of controlled medications and surgical class. We used descriptive analysis to summarize each variable as mean or frequency for continuous and categorical variables and subsequently assessed the association between demographic variables and drug prescription and surgical class. Of the 378 patients who met our inclusion criteria, 68.0% were females and 32.0% were males. The average age was 77.8 years. Most patients had a displaced hip fracture (61%). Most patients underwent a hip hemiarthroplasty (233, 61.6%) versus either a closed reduction with percutaneous pinning (125, 33.1%) or hip open reduction internal fixation (20, 5.3%). There was no significant difference between polypharmacy and hip fracture surgery; however, reported alcohol use was significant in both groups. In our patient population, opioid and/or benzodiazepine prescriptions were not significantly linked to hip fracture surgery, but documented alcohol use was found to be significant in both groups. We noted a higher prevalence of opioid and benzodiazepine prescriptions than was previously reported. As patients age, we should be cautious about the effects of polypharmacy and alcohol use and their impacts on the elderly.

## 1. Introduction

With the growth of the aging population in the United States, the incidence of femoral neck (hip) fractures has increased accordingly.^[1,2]^ Falls are among the most common reasons for hip fractures in the elderly.^[3]^ Each year, approximately 36 million older people experience documented falls, leading to over 32,000 fatalities.^[4]^ Within this annual toll, at least 300,000 elderly individuals find themselves hospitalized, with over 95% of these hospitalizations being due to hip fractures from falls.^[4,5]^ The most common mechanism of fracture is a fall among the elderly.^[6]^ It is noteworthy that women experience falls more frequently than men, constituting 3-quarters of all reported hip fractures.^[4]^ The fall rate per 100 discharges in older adults aged 65 and greater was 53.0, while the mortality rate for those who experienced falls was 33.2.^[7]^ Complications resulting from falls, the leading cause of death from injury in men and women older than age 65, increases in incidence among those over 64 years old and vary according to living status.^[8]^

### 1.1. Polypharmacy and fall risk

A systematic review and meta-analysis published in 2022 found that “Older age, polypharmacy, malnutrition, frailty, smoking and alcohol consumption increased the risk of falls.”^[9]^ Falls can be precipitated by a wide variety of drugs, such as cardiovascular medications, antidepressants and antipsychotics, benzodiazepines, and opioids.^[10]^ The use of 1 or more psychoactive medications is also among the factors associated with the increased risk of fall in the elderly.^[9,11]^ Helgadóttir et al^[12]^ found that an elderly person having even more than 1 prescription led to a 2-fold increased risk of falls which resulted in subsequent hospitalizations. Due to the escalating burden of chronic illnesses within the elderly population, there is a heightened susceptibility to polypharmacy.^[9]^ Seppala et al^[13]^ showed that the initiation of opioids was associated with falls (odds ratio [OR] = 1.51 [1.14–1.99]), as was prescription of benzodiazepines (OR = 1.42 [1.22–1.65]).^[14]^ The simultaneous use of benzodiazepines and opioids poses a significant concern.^[15]^ Noncardiac preoperative individuals with concurrent prescriptions for both opioids and benzodiazepines experienced increased higher 30-day mortality rates and a heightened risk of long-term mortality, particularly among those who were prescribed larger quantities of these medications, when compared to matched control patients.^[16]^ Elderly individuals aged 65 and older who use opioids and benzodiazepines in the month leading up to an event are at an increased risk of experiencing falls that result in hip fractures.^[17]^

### 1.2. Hip fracture surgeries

**Figure 1.**
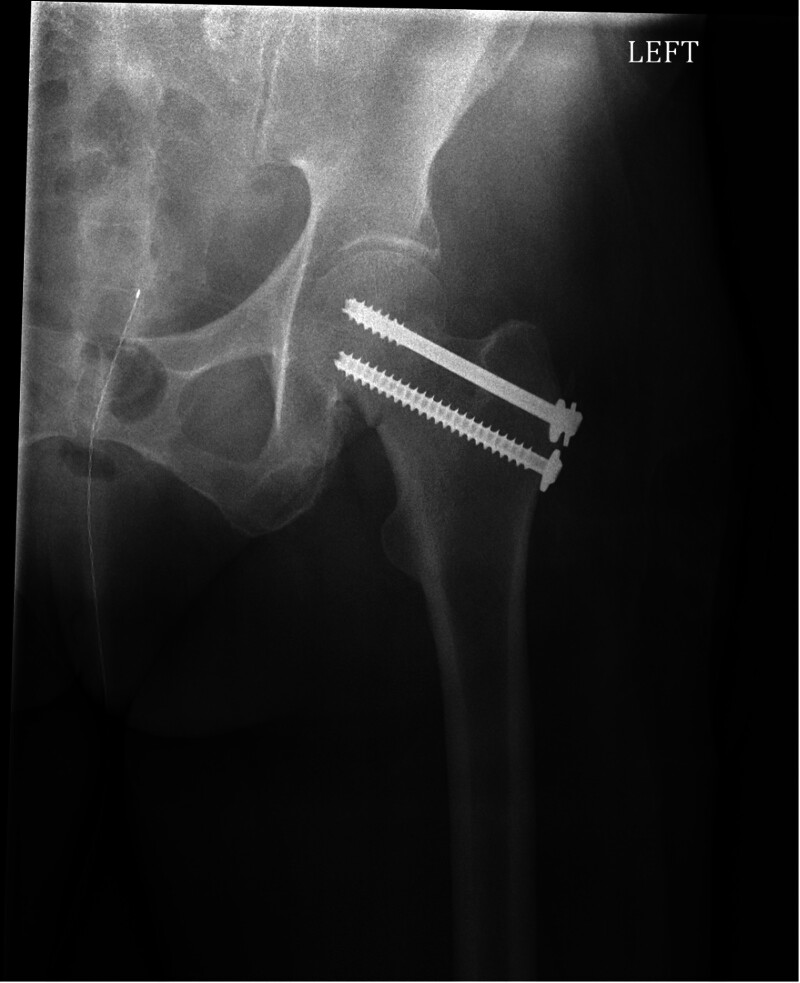
Closed reduction and percutaneous screw fixation.

The subsequent surgeries needed to treat hip fractures have specific risks associated with them. Orthopedic surgeons mainly treat geriatric hip fractures with arthroplasty or closed reduction with internal fixation. Closed reduction and percutaneous pinning (CRPP) involves using fluoroscopy (radiographic machine used in the operating room) and screws to secure the femoral neck fracture (FNF) in the nondisplaced position (Fig. 1). This procedure can be done on nondisplaced FNFs and has minimal blood loss due to the percutaneous/small incision needed to place the screws.^[18]^

**Figure 2.**
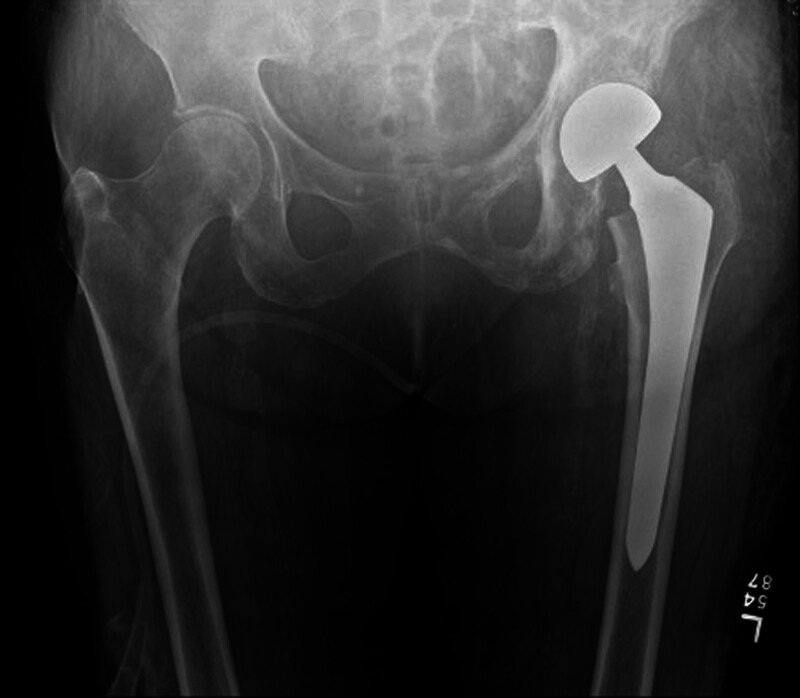
Hip hemiarthroplasty.

The most common type of surgery for a displaced FNF is a hip arthroplasty, with the most common type of arthroplasty being a hemiarthroplasty (Fig. 2). In a hemiarthroplasty a large 10 to 15 cm incision is made on the lateral side of the hip and buttocks through which the broken femoral neck is accessed. Once the FNF is accessed the broken part of the neck attached to the head is removed and sized with measuring devices in the operating room. The femoral neck that remains attached to the femur is shaved down with a saw. Once the femoral neck is shaved and the femoral head sized, a metal stem is placed into the femur and the sized femoral head is placed on the top of this stem. After the stem and the head are assembled, the hip is reduced so that the femoral head sits in the acetabulum.

**Figure 3.**
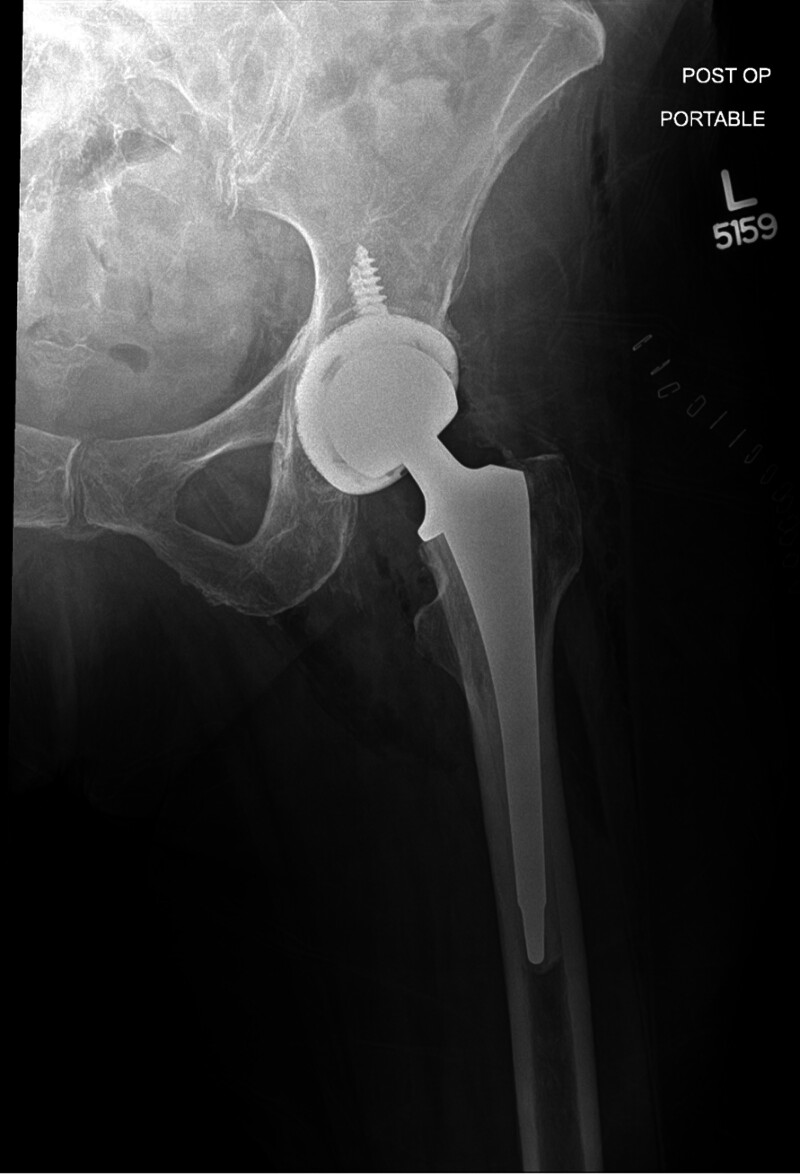
Total hip arthroplasty.

A total hip arthroplasty can also be performed for FNF, but adds a third step where the acetabulum or the cup side of the hip joint is also replaced with a new metal and plastic cup (Fig. 3).^[18]^

There is some evidence that elderly patients with proximal femur fractures treated with hemiarthroplasty have improved healthcare-related quality of life compared to those treated with hip open reduction internal fixation (ORIF).^[19]^ However, a randomized controlled trial evaluating a patient’s ability to return to prior hip function after a nondisplaced FNF, hemiarthroplasty was not superior to internal fixation.^[20]^ Hemiarthroplasty was also associated with increased operative times, increased intraoperative blood loss, and increased hospital length of stay in nondisplaced FNF population.^[20]^ Further evidence supports that preoperative use of either opioids, benzodiazepines, or both, can drastically impact outcomes such as hospital length of stay, balance, and risk of respiratory depression and cardiac insults.^[21]^

Our study evaluates the characteristics of patients who underwent operations for hip fractures based on both medication prescribed (opioids, benzos, both, or none) and surgical class (CRPP and hip ORIF vs hip hemiarthroplasty). Machado-Duque et al^[17]^ found an association between falls in the elderly with hip fractures and co-prescription; yet there is no literature in the United States analyzing this same relationship. Therefore, the goal of our study is to analyze the effect of opioid and benzodiazepine medication prescriptions on hip fracture surgeries in a population of Americans at a single teaching institution. Our hypothesis is that patients who are on opioids and/or benzo prescriptions prior to sustaining a hip fracture are more likely to have a more complex surgery (hip hemiarthroplasty).

## 2. Methods

The University of Texas Medical Branch Institutional Review Board (FWA#: 00002729) reviewed our study and declared it to be exempt from review by the IRB as this was secondary research for which consent is not required. We conducted a retrospective chart review of patients at a single teaching institution in Galveston County, Texas, USA over a 5-year period (January 1, 2015–December 31, 2020). As such, we waived informed consent. We identified participants in the electronic medical record by the presence of an FNF (hip fracture) treated with surgery, either with hip hemiarthroplasty or with internal fixation, using current procedural terminology codes 27235 and 27236. Inclusion criteria consisted of patients over the age of 18, sustaining a hip fracture during the study period, and having accessible 30-day pharmacy data. Exclusion criteria consisted of those subjects who were <18 years old, who were incarcerated, or who belonged to other vulnerable populations. Cases consisted of patients who had been prescribed an opioid, benzodiazepine, or both classes of medication in the 30 days prior to the date of surgery for hip fracture. Controls consisted of patients who had not been prescribed such medications. Given the limited number of subjects, cases, and controls were not matched.

Information which was collected on each subject included medications prescribed (opioid, benzodiazepine, both, or none), surgery class (CRPP, hip ORIF, or hip hemiarthroplasty), laterality of hip fracture, smoking history, dual x-ray absorptiometry (DEXA) scan completion, fall history, alcohol use history, gender, insurance (as a surrogate for socioeconomic status), age, body mass index (BMI), and the use of any assistive devices for ambulation.

We analyzed the presence of opioid, benzodiazepine, or both medication prescriptions 30 days prior to the date of surgery for hip fracture as well as surgery class (CRPP and hip ORIF vs hip hemiarthroplasty). Hospital records were reviewed for each patient who had a hip fracture. We were able to isolate a 30-day period prior to the injury as the cutoff for inclusion of medication prescriptions in the study. If any information was incomplete or not available for a patient, we excluded that patient from the final analysis. As charts were reviewed retrospectively and we included all patients with complete information, we eliminated potential sources of bias. We analyzed data for 378 hip fractures.

We carried out a descriptive analysis summarizing each variable as mean (standard deviation) or frequency (percentage) for continuous and categorical variables, respectively. We assessed the association between demographic factors and drug prescriptions (none, benzodiazepine, opioid, both), and surgery class using *t* test or Chi-squared test/Fisher exact test, for continuous and categorical variables, respectively. Finally, we carried out a logistic regression for medication prescription for surgery class, laterality, smoking, DEXA scan, alcohol, insurance, and assistive device use. We set statistical significance at a *P* value ≤.05. For missing variables, we noted it as such during data collection and excluded the missing variables during the final analysis.

## 3. Results

We screened 385 fractures of which 378 fractures met the inclusion criteria. Women sustained 257 (68.0%) fractures and men sustained 121 (32.0%) fractures. Average age of the patients was 77.8 years (±12.6 years). Average BMI was 24.6 kg/m^2^ (±5.2 kg/m^2^). Those with commercial insurance (medicare or commercial payor) sustained 353 fractures (93.3%) and those that were not commercially insured (medicaid or self-pay) sustained 25 (6.6%) fractures. Three hundred five (79.2%) had no documented history of smoking, but 73 (19.0%) either currently smoked or were former smokers. Two hundred fifty-five (66.2%) patients identified as being teetotalers, while 123 (31.9%) were consumers of alcohol. Eighty-five (22.5%) patients completed a DEXA scan, while the majority 293 patients (77.5%) did not have one completed. Several patients used an assistive device including rolling walkers (126 patients, 33.3%), cane (74 patients, 19.6%), walker (16, 4.2%), wheelchair (45 patients, 11.9%), 4-wheeled walker with seat (1 patient, 0.3%), scooters (4 patients, 1.1%), and crutches (5 patients, 1.3%). One hundred fifteen patients were on either benzodiazepine (24, 6.3%), opioids (77, 20.4%), or both prescribed medication classes (14, 3.7%) prior to their fracture (115 patients, 30.4%). Most prescribed opioids were tramadol (57, 15.1%), hydrocodone-acetaminophen (34, 9.0%), acetaminophen-codeine (12, 3.2%), morphine (9, 2.4%), hydromorphone (2, 0.5%), and oxycodone-acetaminophen (1, 0.3%). Most prescribed benzodiazepines were clonazepam (12, 3.2%) lorazepam (10, 2.6%), alprazolam (8, 2.1%), diazepam (3, 0.8%), temazepam (3, 0.8%), triazolam (1, 0.3%), and estazolam (1, 0.3%). Only 10 patients (2.6%) had a documented history of a fall upon chart review. Patients sustained both left (198, 52.4%) and right (180, 47.6%) hip fractures. Most patients underwent a hip hemiarthroplasty (233, 61.6%) versus either a closed reduction with percutaneous pinning (125, 33.1%) or hip ORIF (20, 5.3%).

Initial bivariate analysis evaluated the groups based on medication prescribed (none, benzodiazepine only, opioid only, or both medication classes). There was a statistically significant difference between the groups with the categories of DEXA (*P* = .005), documented alcohol use (*P* = .021), and wheelchair use (*P* = 0). There was a significant difference in the odds of having a DEXA scan between patients who had controlled medication use compared to those who did not (OR = 2.411, 95% confidence interval [CI] [1.410–4.122]). There was a significant difference in the odds of alcohol use between patients who had controlled medication use compared to those who did not (OR = 2.047, 95% CI [1.249–3.364]). There was a significant difference in the odds of wheelchair use between patients who had controlled medication use compared to those who did not (OR = 3.014, 95% CI [1.546–5.935]). Table 1 describes the characteristics of patients by drug category.

**Table 1:** Characteristics by drug category.

	None	Benzo	Opioid	Both	*P* value
Surgery					
CRPP	76	10	33	6	.2
Hip ORIF	16	1	2	1	
Hip hemiarthroplasty	171	13	42	7	
Surgery class					
Hip hemi	171	13	42	7	.233
CRPP + ORIF	92	11	35	7	
Laterality					
Left	132	14	45	7	.57
Right	131	10	32	7	
Smoking					
No	217	20	57	11	.4
Yes	46	4	20	3	
DEXA					
No	217	16	51	9	.005
Yes	46	8	26	5	
History of fall?					
No	257	24	73	14	.502
Yes	6	0	4	0	
Alcohol					
No	190	15	42	8	.021
Yes	73	9	35	6	
Gender					
Female	182	20	45	10	.11
Male	81	4	32	4	
Ethnicity					
Hispanic or Latino	30	2	9	0	.702
Not Hispanic or Latino	233	22	67	14	
Race					
Black	15	1	13	0	.058
White	244	23	62	14	
Other	4	0	2	0	
Insurance					
Commercial	246	24	70	13	.451
Noncommercial	17	0	7	1	
Assistive devices					
Rolling walker					
No	182	15	47	8	.418
Yes	81	9	30	6	
Cane					
No	212	19	60	13	.685
Yes	51	5	17	1	
Walker					
No	251	24	73	14	.821
Yes	12	0	4	0	
Wheelchair					
No	243	23	57	10	0
Yes	20	1	20	4	
4 wheeled					
No	263	23	77	14	.101
Yes	0	1	0	0	
Scooter					
No	259	24	77	14	.724
Yes	4	0	0	0	
Crutches					
No	260	24	76	13	.296
Yes	3	0	1	1	
Age					
Mean	77.67	75.96	78.7	78.0	
SD	12.77	12.51	11.76	14.23	
N	263	24	77	14	
*R*		−1.707	1.036	0.335	
*P* value		.526	.527	.923	
BMI					
Mean	24.44	24.69	24.84	25.78	
SD	4.67	6.67	5.57	9.13	
N	247	24	77	13	
*R*		0.247	0.397	1.334	
*P* value		.825	.561	.370	

BMI = body mass index, CRPP = closed reduction and percutaneous pinning, DEXA = dual x-ray absorptiometry, ORIF = open reduction internal fixation, SD = standard deviation.

Another bivariate analysis evaluated the groups based on surgery performed (CRPP and hip ORIF vs hip hemiarthroplasty). There was a statistically significant difference between the groups within the categories of documented smoking (*P* = .007), documented alcohol use (*P* = .004), insurance (*P* = .021), and age (*P* = 0). Logistic regression analysis showed statistical significance for documented alcohol consumption and age when evaluating surgery class. There was a significant difference in odds of alcohol use between patients who had hip hemiarthroplasty versus CRPP or hip ORIF (OR = 0.581, 95% CI [0.364–0.928]). There was also a significant difference in age with an OR of 1.049, 95% CI (1.028–1.073) between the 2 surgical groups. Table 2 describes the characteristics of patients by surgery class.

**Table 2:** Characteristics by surgery class.

	CRPP + hip ORIF	Hip hemiarthroplasty	*P* value
Laterality			
Left	82	116	.2
Right	63	117	
Smoking			
No	107	198	.007
Yes	38	35	
DEXA			
No	112	181	.92
Yes	33	52	
History of fall			
No	141	227	1
Yes	4	6	
Alcohol			
No	85	170	.004
Yes	60	63	
Gender			
Female	95	162	.416
Male	50	71	
Ethnicity			
Hispanic	17	24	.648
Not Hispanic	127	209	
Race			
Black	10	19	.814
White	132	211	
Other	3	3	
Insurance			
Commercial	130	223	.021
Noncommercial	15	10	
Assistive devices: rolling walker			
No	98	154	.765
Yes	47	79	
Assistive devices: cane			
No	121	183	.242
Yes	24	50	
Assistive devices: walker			
No	140	222	.55
Yes	5	11	
Assistive devices: 4-wheeled walker with seat			
No	145	232	1
Yes	0	1	
Assistive devices: scooter			
No	144	230	1
Yes	1	3	
Assistive devices: crutches			
No	141	232	.074
Yes	4	1	
Age			
Mean	73.09	80.7	
SD	15.14	9.61	
N	145	233	
*R*		7.610	
*P* value		.0000	
BMI			
Mean	24.56	24.61	
SD	5.44	5.07	
N	135	226	
*R*		0.049	
*P* value		.931	

BMI = body mass index, CRPP = closed reduction and percutaneous pinning, DEXA = dual x-ray absorptiometry, ORIF = open reduction internal fixation, SD = standard deviation.

Although smoking and insurance, as a surrogate for socioeconomic status, were significant in the bivariate analysis, this did not carry forward in the multivariate analysis by surgical class. For smoking, the OR = 0.901, 95% CI (0.502–1.633), and for insurance status it was OR = 1.208, 95% (0.452–3.203).

## 4. Discussion

Contrary to our hypothesis, there were no significant differences identified in our analysis with medication use and hip fracture surgery class. Only alcohol had significance in our analysis when evaluating both medication use and surgery class.

### 4.1. Alcohol use

Persons who had documented alcohol use were less likely (OR = 0.581) to have a hip hemiarthroplasty than having a CRPP or hip ORIF. Cauley et al^[22]^ found in their study that alcohol consumption decreased displaced FNFs which required hemiarthroplasty for surgical treatment. This is consistent with the findings of our study that persons who had documented alcohol use were less likely to have a hip hemiarthroplasty, used to treat a displaced FNF, compared to a CRPP, used to treat a nondisplaced fracture.

Among the findings, 22.5% of our study population’s charts documented that the subject consumed alcohol. One may speculate that alcohol consumption may increase risk of fall and subsequent severity of fracture necessitating a more complex surgery.^[3]^ The odds of having documented alcohol use were 2.047 times greater among those who were on controlled medication rather than those not on controlled medications, which is consistent with the literature.^[9]^

### 4.2. Patient demographics

In our study population, women sustained more fractures than men with an average age of 77.78 years, which is consistent with known hip fracture literature.^[3]^ Surprisingly, the average BMI was 24.6 kg/m^2^ (high end of normal weight) which contrasts with the literature which shows that a higher BMI is a protective factor in hip fractures.^[23]^ Older patients were more likely to have a hip hemiarthroplasty compared to younger patients who were more likely to have a CRPP or hip ORIF. This finding is also consistent with the literature which found that there was an increase in rate of displaced FNFs which are treated with a hemiarthroplasty as person ages.^[22]^

### 4.3. Use of assistive devices

Several patients used assistive devices to ambulate, which may be secondary to poor balance at baseline and predisposing them to hip fractures.^[3]^ The odds of having wheelchair use were 3.014 times greater among those who were on controlled medication rather than those who did not have controlled medication. Studies have reported that the rate of falling from a wheelchair is as high as 57.4%.^[24]^ The known sedating effect of controlled medications may be a contributing factor in the falls of the wheelchair users compared to non-wheelchair users.

### 4.4. Prevalence of prescriptions

The prevalence of opioid prescriptions, benzodiazepine prescriptions, and co-prescription of opioid and benzodiazepine medications in this cohort with hip fractures was slightly higher than was previously reported.^[17]^ Table 3 details the drug classes prescribed in present study compared to study by Machado-Duque et al.^[17]^

**Table 3:** Drug classes prescribed in present study compared to study by Machado-Duque et al.^[8]^

	Present	Machado-Duque et al^[9]^
Opioid	20%	12.70%
Benzodiazepine	6.20%	4.20%
Both	3.60%	

### 4.5. DEXA scans

Despite grade B recommendations by the United States Preventive Services Task Force, most of our study population did not have a DEXA scan within our health records.^[25]^ The odds of having a DEXA scan among those who were prescribed controlled medications were 2.411 times greater than those not on opioid medications. The screening rate among the female medicare participants is only 9.5%.^[26]^ Due to increased regulation of controlled medication use requiring more interactions with the health care system, the subjects on controlled medications may have been more likely to be counseled on getting a DEXA scan than those who had less frequency of visits.^[27]^

### 4.6. Future research and limitations

Reports and practice have shown that other medications aside from opioids and benzodiazepines can contribute to falls. Although it is widely reported, it would be difficult to conclude that these medications were causative of falls or hip fractures in this patient population. We would need to have either a serum or drug test done at the time of fall to evaluate a more definitive relationship. The inclusion of younger patients in this study was important because if these medications do contribute to falls, alterations in bone mineral density, or other confounders, then they would affect the younger patient population as well as the elderly population that is more susceptible to hip fracture at baseline. Comparison of these variables with a large cohort of younger and older patients would be an area of future study.

Future research may also involve prospective review of patients looking specifically at what combinations of opioids and benzodiazepines they are taking, the frequency, and confirmation that the patient is consuming the drugs. With the wide variety of half-lives and side effects, it might be discovered that certain combinations of these drugs are worse than others. Also, future research might look at the treatment of these patients not only for the hip fracture they sustained, but also the need for prophylactic fixation of the nonimpacted side. Previous studies have also looked at the impact of general versus spinal anesthesia, which may also be a future consideration for investigation.^[28]^ If a patient has proven to fall and have a complex high-energy injury, it might be beneficial to prophylactically fix the other side which would prevent fracture.

This study has limitations. The retrospective design has inherent restrictions that occur from the availability of the data. Also, the way in which the data were documented was a dependent perspective. We believe that the accuracy of our primary variable in this case was controlled because of the use of electronic prescribing for controlled medications. Also, we only evaluated that a medication was prescribed to the patient 1 month before the surgery. We were not able to discern what the patient was consuming and which medications, if any, were present at the time that the patient sustained the hip fracture. A drug test at the time of initial evaluation may be better able to ascertain medications that may have contributed to the fall. We were unable to control for the presence of other medications that patients were taking that could have contributed to their hip fracture, such as cardiovascular medications or medications for managing osteoporosis. Patients may have also been on nonprescribed medications and may be misclassified in the nonmedication use category. We had to rely on documentation by multiple clinicians regarding history of falls and may have been subject to incomplete documentation. Mechanism of injury would be important information to have and is a possible confounder but was difficult to ascertain from chart review. Lastly, we did not calculate the morphine equivalents per day to standardize the opioid effect from different medications, as the primary objective was to find a relationship before further stratification.

## 5. Conclusion

Monitoring the use of the opioid and benzodiazepine classes of drugs in managing patient comorbidities is important for the prescribing physician. It is difficult to generalize these drugs as noncontributory to hip fracture surgery, given the scope of patients that were evaluated. Patients who fall and require surgical management for fracture must be evaluated on a case-by-case basis. As patients age, we should be cautious about the effects of polypharmacy and alcohol use and their impacts on the elderly.

## Acknowledgments

Christen Walcher and Jennifer Edwards in their help with manuscript editing.

## Author contributions

**Conceptualization:** Andrew Z. Coskey, Haris Vakil, Namita Bhardwaj.

**Data curation:** Andrew Z. Coskey, Ernst J. Nicanord, Margaret A. Goodwin, Haris Vakil, Daniel C. Jupiter, Namita Bhardwaj.

**Formal analysis:** Andrew Z. Coskey, Ernst J. Nicanord, Margaret A. Goodwin, Haris Vakil, Daniel C. Jupiter, John C. Hagedorn II, Namita Bhardwaj.

**Investigation:** Andrew Z. Coskey, Daniel C. Jupiter, John C. Hagedorn II, Namita Bhardwaj.

**Methodology:** Andrew Z. Coskey, Margaret A. Goodwin, Haris Vakil, Daniel C. Jupiter, Namita Bhardwaj.

**Project administration:** Andrew Z. Coskey, Margaret A. Goodwin, Haris Vakil, Daniel C. Jupiter, John C. Hagedorn II, Namita Bhardwaj.

**Supervision:** Andrew Z. Coskey, Ernst J. Nicanord, Daniel C. Jupiter, John C. Hagedorn II, Namita Bhardwaj.

**Validation:** Andrew Z. Coskey, Daniel C. Jupiter.

**Writing—original draft:** Andrew Z. Coskey, Ernst J. Nicanord, Margaret A. Goodwin, Haris Vakil, Daniel C. Jupiter, John C. Hagedorn II, Namita Bhardwaj.

**Writing—review & editing:** Andrew Z. Coskey, Ernst J. Nicanord, Margaret A. Goodwin, Haris Vakil, Daniel C. Jupiter, John C. Hagedorn II, Namita Bhardwaj.

**Resources:** Daniel C. Jupiter.

**Visualization:** Daniel C. Jupiter.
